# Relationship between Cognitive and Sleep–wake Variables in Asymptomatic Offspring of Patients with Late-onset Alzheimer’s Disease

**DOI:** 10.3389/fnagi.2017.00093

**Published:** 2017-04-05

**Authors:** Carolina Abulafia, Bárbara Duarte-Abritta, Mirta F. Villarreal, María S. Ladrón-de-Guevara, Celeste García, Geraldine Sequeyra, Gustavo Sevlever, Leticia Fiorentini, Karl-Jürgen Bär, Deborah R. Gustafson, Daniel E. Vigo, Salvador M. Guinjoan

**Affiliations:** ^1^FLENI Foundation Department of PsychiatryBuenos Aires, Argentina; ^2^Applied Neuroscience Laboratory, Institute for Biomedical Research, School of Medical Sciences, Universidad Católica ArgentinaBuenos Aires, Argentina; ^3^Consejo Nacional de Investigaciones Científicas y TécnicasBuenos Aires, Argentina; ^4^Department of Psychiatry and Psychotherapy, Universitätsklinikum Jena, Friedrich-Schiller-UniversitätJena, Germany; ^5^Department of Neurology, State University of New York - Downstate Medical Center, BrooklynNY, USA; ^6^Neuropsychiatric Epidemiology Unit, University of GothenburgGothenburg, Sweden; ^7^Department of Health and Education, University of SkövdeSkövde, Sweden; ^8^FLENI Teaching Unit, Department of Psychiatry and Mental Health, University of Buenos Aires School of MedicineBuenos Aires, Argentina; ^9^Department of Neurophysiology, University of Buenos Aires School of PsychologyBuenos Aires, Argentina

**Keywords:** early diagnosis, late-onset Alzheimer’s disease, circadian rhythms, cardiac autonomic control, actigraphy

## Abstract

Early neuropathological changes characteristic of late-onset Alzheimer’s disease (LOAD) involve brain stem and limbic structures that regulate neurovegetative functions, including sleep–wake rhythm. Indeed, sleep pattern is an emerging biomarker and a potential pathophysiological mechanism in LOAD. We hypothesized that cognitively asymptomatic, middle-aged offspring of patients with LOAD (O-LOAD) would display a series of circadian rhythm abnormalities prior to the onset of objective cognitive alterations. We tested 31 children of patients with LOAD (O-LOAD) and 19 healthy individuals without family history of Alzheimer’s disease (control subjects, CS) with basic tests of cognitive function, as well as actigraphy measures of sleep–wake rhythm, cardiac autonomic function, and bodily temperature. Unexpectedly, O-LOAD displayed subtle but significant deficits in verbal episodic memory (Rey Auditory Verbal Learning Test delayed recall 10.6 ± 0.4 vs. 8.6 ± 0.6, *t* = 4.97, *df* = 49, *p* < 0.01) and language (Weschler’s vocabulary 51.4 ± 1.3 vs. 44.3 ± 1.5, *t* = 2.49, *df* = 49, *p* < 0.001) compared to CS, even though all participants had results within the clinically normal range. O-LOAD showed a phase-delayed rhythm of body temperature (2.56 ± 0.47 h vs. 3.8 ± 0.26 h, *t* = 2.48, *df* = 40, *p* = 0.031). Cognitive performance in O-LOAD was associated with a series of cardiac autonomic sleep–wake variables; specifically indicators of greater sympathetic activity at night were related to poorer cognition. The present results suggest sleep pattern deserves further study as a potential neurobiological signature in LOAD, even in middle-aged, at risk individuals.

## Introduction

Alzheimer’s disease (AD) is a progressive neurodegenerative disorder that causes up to 80% of all dementia cases worldwide (Alzheimer’s Association, 2016). It is characterized by the coexistence of two neuropathological hallmarks, namely extracellular plaques of amyloid beta (Aβ) and intracellular neurofibrillary tangles made up of hyperphosphorylated Tau protein (Tau). Severe neurodegeneration and widespread neuroinflammation are ubiquitous albeit less specific neuropathological features of the disorder. Over 99% of AD cases are late-onset (i.e., the initial cognitive symptoms usually appear after 65 years of age); familial or early-onset AD cases result from fairly rare autosomal dominant mutations, and account for less than 1% of cases, including the patient of the original description of the disorder (Alzheimer, 1907). The relationship between Aβ- and Tau-related pathology in AD is complex and remains elusive in its details to this day. The amyloid cascade hypothesis (ACH) posits that AD begins with the deposition of Aβ, resulting from increased production, reduced clearance, or a combination or both. The deposition of Aβ would in turn trigger a cascade of neurodegenerative processes including Tau-related intracellular changes, loss of synapses, inflammation neurodegeneration, and eventually regional brain atrophy (Hardy and Higgins, 1992). The ACH got support from evidence that familial forms of AD were due to dominant mutations in enzymes participating in the metabolism of amyloid. Down syndrome [resulting in an extra copy of the gene coding for the amyloid precursor protein (APP) located in chromosome 21], which is associated with increased incidence and earlier onset of AD, lent further support to the ACH. However, the main conceptual problem of this hypothesis is a remarkable lack of correlation between Aβ deposits and cognitive changes, as well as the absence of a clear and orderly pattern of anatomical progression as the disease advances. Further, significant *in vivo* Aβ deposition is present in a substantial number of cognitively normal elderly individuals, and it is associated with gray matter (GM) changes not characteristic of the early phases of neurodegeneration of AD (Sepulcre et al., 2016). In spite of this evident lack of correlation between Aβ deposition and cognitive symptoms, a number of pharmacological agents targeting Aβ accumulation have failed or even resulted in worse outcomes than placebo. In fact, this group of experimental drugs (including anti-Aβ antibodies seeking to improve clearance of brain amyloid, and drugs interfering with the synthesis of new amyloid) have met with one of the highest failure rates in any therapeutic area in the history of medicine (Cummings et al., 2014).

On the other hand, Tau pathology in AD follows a highly predictable progression, predates Aβ accumulation in anatomical studies, and shows a parsimonious relationship with cognitive symptom development (Braak and Del Tredici, 2011; Walsh et al., 2017) being the basis for the most accepted staging system of AD pathology (Braak and Braak, 1991).

Early detection of cases of AD, before any cognitive symptoms emerge, is a challenge as important as discovering treatments aimed at the causes of AD. This should be accomplished before neuropathological changes are so advanced that they cannot be reversed. In fact neuropathological studies suggest changes characteristic of AD occur already in the third and fourth decades of life, involving phosphorylated Tau accumulation in selected groups of neurons, including those in the locus coeruleus, and diencephalic and allocortical limbic areas (Braak and Del Tredici, 2011; Buchhave et al., 2012). Such areas are involved in the regulation of vegetative functions, including autonomic nervous system regulation and diurnal variation (Braak et al., 2011). Sleep–wake cycle regulation in particular, depends on intrinsic circadian rhythm generation by diencephalic structures, in addition to a series of environmental cues of sleep–wake cycle (Buijs et al., 2016). Thus, alterations in sleep could represent, in theory, early manifestations of late-onset AD (LOAD)-related neuropathological changes in the brain.

Sleep abnormalities have emerged as potential early biomarkers of LOAD status (see Mander et al., 2016 for a review on this topic). AD-related changes in sleep have also been proposed as possible neuropathogenic mediators that perpetuate the cycle of amyloid deposition, neurofibrillar changes, and neurodegeneration ultimately resulting in cognitive and functional deterioration (Lim et al., 2013; Mander et al., 2015). In addition, previous evidence suggests that in cognitively asymptomatic, middle-aged individuals, amyloid deposition is associated with poor sleep (Sprecher et al., 2015).

We hypothesized that circadian alterations could be an early functional manifestation of AD neuropathology in at-risk individuals. To this end, we studied a group of cognitively normal, middle-aged individuals with at least one parent diagnosed with LOAD, which accounts for over 99% of all cases of AD (Dubois et al., 2016). We specifically predicted that cognitively asymptomatic offspring of LOAD patients (O-LOAD) would display abnormalities in sleep, circadian autonomic activity and diurnal variation, and circadian rhythm of body temperature as compared with healthy individuals without a family history of AD. Moreover, we expected to find correlations between such circadian alterations (in the form of decreased amplitude of diurnal variation, phase alterations, sleep efficiency, and greater sympathetic or lesser parasympathetic activity), and cognitive performance in verbal memory in O-LOAD but not healthy individuals.

## Materials and Methods

### Design and Sample

This was a cross-sectional study, where cognitive and chronobiological measures were compared between a sample of AD offspring (O-LOAD) and control subjects (CS). The study protocol was performed in accordance with the Declaration of Helsinki, and approved by the Bioethics Committee of FLENI Foundation, Argentina. All participants provided their written informed consent for the study.

A total of 31 O-LOAD participated in the study along with 19 healthy subjects with no family history of AD (CS), consecutively selected and comparable in gender, age, and education level. The inclusion criteria for O-LOAD were as follows: (1) To have at least one parent diagnosed with probable LOAD according to the DSM-5, (2) to be 40–65 years old at the time of recruitment, and (3) seven or more years of formal education. The CS group had the same inclusion criteria except for item 1). The exclusion criteria for O-LOAD and CS were as follows: (1) Mini Mental State Examination (MMSE) score <25, (2) compromised intellectual level based education and employment history, (3) evidence of current progressive neurologic disease or likely to impair cognitive performance, (4) history of substance abuse (alcohol, marijuana, stimulants, benzodiazepines, or other drugs), and (5) Hachinski score >7 (to filter data suggestive of vascular-derived cognitive impairment).

### Cognitive Assessment

The neuropsychological tests selected for this study have been widely validated and are frequently used in clinical practice and thus require no detailed explanation. They comprise a concise battery aimed at assessing the most salient cognitive domains impaired in AD: episodic memory and language. Neuropsychological evaluation was performed in a single session of approximately 90 min by an experienced neuropsychologist (CA). All evaluations were performed between 12.00 and 17.00 h. The MMSE (Folstein et al., 1975) and the Clock Drawing Test subtest from the 7 Minute Screen test (Solomon et al., 1998) were included as screening measurements; whereas MMSE is insensitive to even prodromal AD, we incorporated it as a widely used screening instrument (Ravaglia et al., 2005), to be complemented with more sensitive tests. Thus, verbal episodic memory was assessed by the Rey Auditory Verbal Learning Test (RAVLT; Rey, 1964; Schmidt, 1996). Semantic memory, or “the knowledge of words” was measured by the semantic fluency task (“animals” category) (Spreen and Benton, 1969) and the vocabulary subtest of the intelligence battery WAIS-III (Wechsler, 1997). Additionally, the Cognitive Reserve Questionnaire (CRQ) was administered to all participants. This technique proves to be quick and useful screening to assess the most relevant elements associated to the cognitive reserve (education level, parent’s education level, additional academic courses completed, professional activity, musical education, fluent languages, reading activity, and ingenuity games) (Rami et al., 2011). Finally, the presence and severity of depressive symptoms was measured by the Beck Depression Inventory (self-report) and by the Hamilton Depression Rating Scale (HDRS) completed by a clinician.

All participants were cognitively asymptomatic and their neuropsychological testing yielded normal values, so none of the individuals met criteria for mild cognitive impairment or dementia.

### Assessment of Neurovegetative Function

Two questionnaires were used to measure quality of sleep: Pittsburgh Sleep Quality Index (PSQI) and Epworth Sleepiness Scale (ESS). The PSQI is a self-report questionnaire that assesses sleep problems in the last month. A PSQI score greater than 4 points is defined as “poor sleep quality.” The ESS measures daytime sleepiness. An ESS score greater than 10 represents “excessive daytime sleepiness” (Diez et al., 2011).

Objective evaluation of the sleep–wake cycle was performed by actigraphy. Subjects wore the actigraph (MicroMini-Motionlogger, Ambulatory Monitoring Inc, NY, USA) on their wrist for 7 days. During this time, they were also requested to complete a Daily Sleep Diary where they reported sleep habits including sleep onset and offset. Time spent in bed, time awake, and sleep efficiency were calculated using Action W 2.5 (Ambulatory Monitoring Inc, NY, USA) (Bellone et al., 2016).

Distal skin temperature was measured as a proxy for the circadian rhythm of core temperature. There is an approximate 12-h phase difference between distal skin temperature and core body temperature and both measures behave in the same manner, reaching its maximum levels during the sleeping period and decreasing during waking hours (Sarabia et al., 2008). For this study, 16 mm × 6 mm Thermochron iButton^®^ DS1291H (Dallas Maxim) sensors were placed next to the actigraph. Temperature samples were obtained every 10 min for 7 days (van Marken Lichtenbelt et al., 2006). The Chronos-Fit software was used to fit a cosine curve with a 24-h period to the data as an estimate of the circadian pattern, using the partial Fourier series method. From that model, the following measures were derived: % rhythm: represents the percentage of variation in the data explained by the fitted model; MESOR (midline estimating statistic of rhythm) is a rhythm adjusted mean; amplitude is the difference between the maximum and the MESOR; acrophase is the time at which the maximum of the rhythm occurs (van Marken Lichtenbelt et al., 2006; Refinetti et al., 2007).

Autonomic nervous system circadian rhythm was measured by heart rate variability (HRV) analysis described in detail elsewhere (Vigo et al., 2005, 2010). Fluctuations in heartbeats are cyclical and have different frequencies. High frequency (HF) modifications respond to respiratory mechanisms and are a marker of parasympathetic activity. Low frequency (LF) fluctuations respond to the baroreflex and present sympathetic and parasympathetic influences. There are also very low frequency (VLF) oscillations, which possibly respond to hormonal or thermal oscillations.

Participants underwent a 24-h digital electrocardiogram (ECG) study (Holtech, Servicios Computados SA, Buenos Aires). The data obtained were processed to calculate the time elapsed between R waves (RR intervals). HRV evaluation consisted of time domain and frequency domain analyses. For the time domain analysis, the following measures were calculated: RRM (mean duration of RR intervals in milliseconds) quantifies the mean heart rate, SDNN (standard deviation of RR intervals in milliseconds) represents a coarse quantification of overall variability, and RMSSD (square root of the mean squared differences of successive normal RR) measures short-term heart rate variations. For the frequency domain analysis, the discrete wavelet transform (DWT) was chosen over the traditional fast Fourier transform because it is not affected by discontinuities or non-stationarities. Before applying the DWT, the linear trend and the mean value were subtracted from the signal. Moreover, it was evenly sampled with a frequency of 2.4 Hz through a spline interpolation algorithm and zero padded to the next higher power of two. The signal was analyzed by a six-level wavelet decomposition with a Daubechies four-wavelet function. After undergoing such a decomposition process, wavelet levels A6 and D1–D6 make up the total power (TP, 0–0.5 Hz), wavelet levels A6 and D6 closely represent the VLF band (0–0.0375 Hz), wavelet levels D4–D5 reflect the LF band (0.0375–0.15 Hz), and wavelet levels D2–D3 approximate to the HF band (0.15–0.6 Hz). In DWT, the square of the standard deviation of wavelet coefficients at each level matches the spectral power of that level. The obtained values are formulated as the natural logarithm of TP, HF, LF, and VLF; normalized units of LF [LF/(TP - VLF)/100] and HF [HF/(TP - VLF)/100]; and the ratio between LF and HF (Vigo et al., 2012).

For the circadian analysis, the ECG recording was segmented in 30-min fragments, which were then averaged according to the sleep or wake periods as defined by actigraphy. Furthermore, sleep–wake autonomic differences were calculated. Daytime sleep periods were classified as naps and excluded from the daytime average.

### Structural MRI

MRI images were acquired on a 3 T GE Signa HDxt MRI machine with an eight-channel head coil. A high resolution T1 3D fast SPGR-IR (TR = 6.604 ms, TE = 2.796 ms, TI = 450) image was acquired. Image correction (ASSET) with acceleration factor = 2; acquisition matrix = 256 × 256; FOV = 24 cm; slice thickness = 1.2 mm; 120 axial contiguous slices.

The acquired images were converted from DICOM format to NIFTI using the SPM12 (Wellcome Department of Cognitive Neurology, London, UK) implemented in MATLAB (MathWorks Inc., Sherborn, MA, USA). The CAT12 tool^1^ for the SPM12 was used to process the images (Preprocessing → Segment Data Batch with default parameter values). A quality control of the images derived from the segmentation was carried out and the relative volumes of GM and white matter (WM) were obtained for each subject.

### Statistical Analysis

Continuous variables were summarized by means and standard errors. Categorical variables were summarized as frequencies and percentages. Differences between groups were calculated by using *t*-test for independent samples, and sex differences in groups by means of the chi-square test. Correlations of chronobiological measures with cognitive and clinical data were evaluated using Pearson correlation coefficients. We report two-tailed signification at *p* < 0.05 and also Bonferroni correction for multiple comparisons. All statistical analysis was performed using the SPSS version 22.0 software (SPSS Inc.).

## Results

Both groups were comparable in age (CS = 54.2 ± 2.2 years vs. O-LOAD = 53.8 ± 1.6 years) and sex (CS 73.7% women vs. O-LOAD 71%). **Table 1** shows the demographic and clinical characteristics of O-LOAD and CS. Groups had similar years of education, Hachinski score, and depressive symptoms (**Table 1**).

**Table 1:** Demographic and clinical data.

	CS		O-LOAD			
	Mean or frequency	SE or %	Mean or frequency	SE or %	*t*	*p*
**Demographic and clinical data**					
*n*	19		31			
Education	17.1	1.2	16.3	0.4	10.732	0.564
CRQ	17.2	0.7	15.3	0.7	1.227	0.071
Hachinski score	1.1	0.3	1.2	0.2	1.172	0.667
BDI-II	9.1	1.9	8.8	1.2	0.068	0.903
HDRS	7.9	1.3	8.8	1.3	0.927	0.635
PSQI	5.7	0.9	6.3	0.7	0.431	0.548
ESS	8.9	1.0	9.3	0.8	0.044	0.808
**Neuropsychological tests**					
*n*	19		31			
MMSE	29.3	0.2	28.9	0.2	1.152	0.103
Clock	5.9	0.3	6.0	0.2	0.280	0.806
Vocabulary	51.4	1.3	44.3	1.5	2.486	0.001
Semantic fluency	21.4	1.3	20.7	0.7	3.474	0.644
RAVLT	45.6	2.2	42.9	1.3	2.271	0.293
RAVLT D	10.6	0.4	8.6	0.6	4.974	0.009
**Brain volume**					
*n*	15		27			
RGM (%)	42.83	0.52	42.74	0.31	1.158	0.880
RWM (%)	34.19	0.37	34.46	0.30	0.342	0.582
**Actigraphy**					
*n*	17		28			
Start time (hh:mm)	00:43	00:14	00:00	00:14	0.669	0.097
End time (hh:mm)	07:55	00:14	07:55	00:14	1.096	0.922
Duration (min)	443.91	12.47	473.70	11.79	0.033	0.090
Efficiency (%)	95.85	0.92	95.64	0.46	0.618	0.839
**Distal temperature**					
*n*	15		26			
% rhythm	26.69	3.95	26.40	2.60	0.157	0.952
MESOR	33.03	0.14	32.16	1.15	1.584	0.456
Amplitude (°C)	0.99	0.11	0.96	0.09	0.052	0.841
Acrophase (time)	2.56	0.47	3.80	0.26	2.478	0.031
**HRV**					
*n*	16		23			
**Wake**					
RRM (ms)	778.49	20.66	794.71	19.38	0.096	0.571
SDNN (ms)	59.99	4.66	63.69	4.38	0.107	0.567
RMSSD (ms)	30.15	2.74	34.28	3.57	0.313	0.365
ln VLF (WPC)	14.90	0.07	15.04	0.06	0.016	0.128
ln LF (WPC)	10.30	0.13	10.54	0.13	0.251	0.194
ln HF (WPC)	7.71	0.14	7.87	0.12	0.030	0.407
L/H	15.10	1.14	16.59	1.23	0.212	0.380
LF (nu)	0.16	0.06	0.10	0.03	2.951	0.414
HF (nu)	0.84	0.06	0.90	0.03	2.951	0.414
**Sleep**					
RRM (ms)	902.90	31.37	926.33	25.43	0.100	0.566
SDNN (ms)	57.00	5.74	62.20	4.68	0.463	0.488
RMSSD (ms)	31.42	3.19	34.17	2.58	0.886	0.508
ln VLF (WPC)	15.14	0.07	15.31	0.10	0.762	0.167
ln LF (WPC)	10.30	0.14	10.48	0.09	2.169	0.263
ln HF (WPC)	7.88	0.16	7.97	0.10	2.048	0.606
L/H	12.32	0.85	14.12	1.30	2.340	0.253
LF (nu)	0.22	0.06	0.22	0.06	0.403	0.946
HF (nu)	0.78	0.06	0.78	0.06	0.403	0.946
**Sleep–wake difference**					
RRM (ms)	130.67	26.37	127.99	20.88	0.067	0.937
SDNN (ms)	-1.28	3.56	-2.07	4.14	0.961	0.886
RMSSD (ms)	1.87	2.41	-0.32	3.71	0.723	0.623
ln VLF (WPC)	0.26	0.06	0.26	0.12	2.445	0.997
ln LF (WPC)	0.03	0.08	-0.07	0.14	3.134	0.522
ln HF (WPC)	0.20	0.10	0.11	0.11	1.268	0.806
L/H	-2.98	0.99	-2.68	1.22	0.576	0.850
LF (nu)	0.07	0.07	0.12	0.05	0.109	0.558
HF (nu)	-0.07	0.07	-0.12	0.05	0.109	0.558

*CRQ, Cognitive Reserve Questionnaire; BDI-II, Beck Depression Inventory, second edition; HDRS, Hamilton Depression Rating Scale; ESS, Epworth Sleepiness Scale; PSQI, Pittsburgh Sleep Quality Index; MMSE, Mini Mental State Examination; RAVLT, Rey Auditory Verbal Learning Test learning curve; RAVLT D, Rey Auditory Verbal Learning Test delayed recall; RGM, relative gray matter; RWM, relative white matter; HRV, heart rate variability; RRM, mean duration of RR intervals; SDNN, standard deviation of RR intervals; RMSSD, square root of the mean squared differences of successive normal RR; WPC, power wavelet coefficients corresponding to: VLF, very low frequency; LF, low frequency; HF, high frequency; L/H, relationship between LF and HF.*

Whereas all participants had normal cognitive performance, as a group, O-LOAD showed lower episodic memory as evidenced by performance on the RAVLT (**Table 2**). The WAIS vocabulary item was also lower among the O-LOAD (**Table 2**).

**Table 2:** Correlation coefficients between HRV and neuropsychological measurements.

	SDNN	RMSSD	LF	HF	L/H
**Wake**					
CRQ	ns	ns	-0.459, 0.028	-0.437, 0.037	ns
RAVLT	**0.557, 0.006**	**0.659, 0.001**	**0.523, 0.010**	**0.562, 0.005**	ns
RAVLT D	0.504, 0.014	**0.579, 0.004**	0.489, 0.018	**0.541, 0.008**	ns
**Sleep**					
Clock	ns	ns	ns	ns	-0.452, 0.035
MMSE	ns	ns	ns	ns	-0.524, 0.012
RAVLT	ns	ns	ns	ns	**-0.559, 0.007**
RAVLT D	ns	ns	ns	ns	-0.508, 0.041
**Sleep–wake**					
MMSE	ns	ns	ns	ns	-0.522, 0.013
RAVLT	-0.484, 0.023	-0.445, 0.038	**-0.613, 0.002**	ns	**-0.744, <0.001**
RAVLT D	ns	ns	**-0.575, 0.005**	ns	**-0.713, <0.001**

*Shown for each variable are: *r, p*. CRQ, Cognitive Reserve Questionnaire; MMSE, Mini Mental State Examination; RAVLT, Rey Auditory Verbal Learning Test learning curve; RAVLT D, Rey Auditory Verbal Learning Test delayed recall. In bold are marked *r, p* surviving Bonferroni correction for multiple measurements.*

O-LOAD showed a delayed phase in the circadian rhythm of body temperature compared to CS (**Table 1**). Measures of sleep–wake cycle in the actigraphy, cardiac autonomic variables, and relative GM and WM brain volumes were similar in both groups as well (**Table 1**).

In CS, relative GM volume showed a direct correlation with learning in RAVLT (*r* = 0.769, *p* = 0.001). We did not observe a relationship between GM or WM volume and cognitive performance variables in O-LOAD.

**Table 2** shows significant correlations between HRV and its diurnal variations and cognitive values in O-LOAD. Learning and recall values on the RAVLT displayed robust correlations with a series of cardiac autonomic activity measures and their diurnal variations. In general, greater HRV and a greater night–day difference in HRV resulted in better verbal memory results. More specifically, an increased sympathovagal balance at night was associated with poorer cognitive results on verbal memory, clock drawing and MMSE score (**Table 2**). All associations between frequency-domain HRV and RAVLT variables survived Bonferroni corrections for multiple tests (**Table 2**, bold *r, p*). These associations were not present in CS (not shown). Sleep efficiency measured with actigraphy in O-LOAD was associated with greater cognitive reserve (*r* = 0.495, *p* = 0.007). Such correlations were again not present in CS (not shown).

## Discussion

The main findings of the present study include (1) lower performance in O-LOAD as compared to CS in measures of verbal memory and language, even though performance in both groups falls within the normal range, (2) a delayed phase of the circadian rhythm of body temperature in O-LOAD, (3) the presence of a relationship between sleep efficiency and verbal memory in O-LOAD, and (4) overall HRV, decreased night–day differences in HRV, and increased sympathovagal balance at night are all related to decreased verbal memory in O-LOAD.

The present observations of a relationship between indicators of healthy circadian and neurovegetative function and cognitive abilities in O-LOAD lend support to the hypothesis that sleep characteristics are associated with changes in cognition typical of AD, and that such a relationship is present over a decade prior to the expected onset of cognitive symptoms. This is compatible with the view that ongoing sleep changes, if persistent, could contribute to the appearance of cognitive symptoms characteristic of AD. Further, we observed a phase delay in the diurnal rhythm of body temperature in O-LOAD, pointing to a basic alteration in the circadian system in this group. Moreover, our findings extend this observation to sleep–wake differences in sympathovagal balance, another limbic phenotype with a potential relationship with early AD pathological changes. HRV is a non-invasive measure of cardiac autonomic output, ultimately reflecting complex regulation at the central autonomic network (see Kemp and Quintana, 2013 for a comprehensive discussion on the origins and interpretation of HRV).

In the present study, we observed a relationship between overall HRV and amplitude of the sleep–wake differences of HRV and better cognition in O-LOAD. More specifically, greater sympathetic prevalence [as denoted by increased low frequency/high frequency (L/H)] at night was associated with more widespread poor results in cognition, including not only verbal memory but overall cognition (as reflected in the MMSE score) and visuospatial/executive function (clock drawing score).A potential link between abnormal sleep–wake variation and poor cognition in the present sample of O-LOAD might be the reported association between reduced parasympathetic activity and decreased sleep quality (Burton et al., 2010), which in turn predicts cognitive decline (Mander et al., 2016). Another potential link may be the increased susceptibility to stress (e.g., Stenfors et al., 2016). However, normal anxiety and depression symptomatology in O-LOAD argue against this possibility. Alternatively, recent data suggest that vascular and endocrine changes might mediate the relationship between cardiac autonomic activity and cognition (Kemp et al., 2016). Results in the study by Kemp et al. (2016) might not be easily comparable to the present study, as they studied HRV in 10-min samples and the cognitive test was TMT-B. Moreover, the normal Hachinski score observed in our O-LOAD sample makes a similar pathophysiologic link less likely in our sample. However, other published results emphasize the need to further investigate the mechanisms linking HRV and cognition. In the case of O-LOAD, the hypothesis that limbic changes underlie both cognitive changes and top-down abnormalities in the control of peripheral autonomic function needs to be tested with the help of structural and functional regional brain measurements focusing on structures known to be affected early in AD.

We could not confirm our prediction of unaffected cognition in this middle-aged, asymptomatic sample of O-LOAD patients. Whereas performance in all participants fell in the range considered normal, delayed recall, learning in the 5th trial, and recuperation on the RAVLT were lower in O-LOAD than in CS. This finding was unexpected given that average age was around 53 years, i.e., approximately a decade prior to the age of expected onset of symptoms. This lends support to the hypothesis that significant AD pathology may be present in at-risk individuals several years prior to cognitive symptom onset (Braak et al., 2011). Such structural changes could affect limbic functions as suggested by the associations between cognition and sleep–wake cycle abnormalities described herein. However, brain anatomical and functional correlates of these findings will have to be confirmed in future studies addressing brain volume and indicators of amyloid deposition in regions known to bear the early impact of AD pathology.

The present conclusions are limited by a series of factors. The relatively small number of participants would require that the present findings be confirmed in larger samples; in particular, we might have overlooked differences between groups in variables of limbic functioning due to small sample size. The only available structural measure was total GM and WM in the encephalon, adjusted for intracranial volume. There were no significant differences in these general measures of neurodegeneration, but regional volumes and brain function measurements were not ascertained so, as stated, the meaning of the present clinical and physiological findings in terms of AD pathology remains to be determined. Albeit significant, correlation values suggest limbic phenotypes explain only up to 25% of the variance in cognitive values, leaving a substantial variability explained by other factors. Last, we observed a trend toward a better cognitive reserve in controls compared to O-LOAD (**Table 1**). Albeit not statistically significant, a better cognitive reserve could explain at least in part the better performance in memory and vocabulary in this group, unrelated to the fact of not having a family history of LOAD.

In sum, the present preliminary results extend previous observations of a relationship between sleep structure and cognition in patients with minor and major neurocognitive disorder due to AD, to cognitively asymptomatic, middle-aged offspring of individuals with AD. Further, it extends the observation to a peripheral indicator of the activity of the central autonomic network, and specifically links autonomic activity during sleep with cognitive performance. The relationship of these observations with specific structural alterations in limbic areas and pattern of amyloid deposit in this group remains unsettled and opened to further investigation. Further, the potential utility of variables studied herein as early biomarkers of AD status would necessitate long-term follow-up in order to detect those individuals who progress to the development of clinical characteristics of AD.

## Author Contributions

SMG, DEV, DRG, KJB, and MFV designed the initial project of this manuscript. CA, BDA, MFV, DEV, SMG, GuS, and LF made the final research plan. CA, BDA, MSLDG, CG, and GeS carried out all the experiments. CA, BDA, DEV, MFV, and SMG ran the statistical analyses and made a definitive interpretation of the findings. CA, DEV, and SMG wrote the first version of the manuscript. All authors edited and approved the final version of the manuscript.

## Conflict of Interest Statement

The authors declare that the research was conducted in the absence of any commercial or financial relationships that could be construed as a potential conflict of interest.

## Footnotes

1 http://dbm.neuro.uni-jena.de/cat/
